# Arg188 in rice sucrose transporter OsSUT1 is crucial for substrate transport

**DOI:** 10.1186/1471-2091-13-26

**Published:** 2012-11-21

**Authors:** Ye Sun, John M Ward

**Affiliations:** 1 Department of Plant Biology, University of Minnesota Twin Cities, St. Paul, MN, 55108, USA

**Keywords:** Sucrose transporter, Major facilitator superfamily, Substrate binding, Mutagenesis

## Abstract

**Background:**

Plant sucrose uptake transporters (SUTs) are H^+^/sucrose symporters related to the major facilitator superfamily (MFS). SUTs are essential for plant growth but little is known about their transport mechanism. Recent work identified several conserved, charged amino acids within transmembrane spans (TMS) in SUTs that are essential for transport activity. Here we further evaluated the role of one of these positions, R188 in the fourth TMS of OsSUT1, a type II SUT.

**Results:**

The OsSUT1(R188K) mutant, studied by expression in plants, yeast, and *Xenopus* oocytes, did not transport sucrose but showed a H^+^ leak that was blocked by sucrose. The H^+^ leak was also blocked by β-phenyl glucoside which is not translocated by OsSUT1. Replacing the corresponding Arg in type I and type III SUTs, AtSUC1(R163K) and LjSUT4(R169K), respectively, also resulted in loss of sucrose transport activity. Fluorination at the glucosyl 3 and 4 positions of α-phenyl glucoside greatly decreased transport by wild type OsSUT1 but did not affect the ability to block H^+^ leak in the R188K mutant.

**Conclusion:**

OsSUT1 R188 appears to be essential for sucrose translocation but not for substrate interaction that blocks H^+^ leak. Therefore, we propose that an additional binding site functions in the initial recognition of substrates. The corresponding Arg in type I and III SUTs are equally important. We propose that R188 interacts with glucosyl 3-OH and 4-OH during translocation.

## Background

Sucrose is an important product of photosynthesis, and is the main form of carbohydrate transported in the phloem in most higher plants [1]. Sucrose transporters (SUTs or SUCs) are membrane proteins that facilitate the uptake of sucrose into the cytoplasm [2]. Driven by the electrochemical H^+^ gradient across the membrane, SUT proteins transport both sucrose and H^+^ into the cytoplasm at a ratio of 1:1 [3-5]. Mutagenesis or antisense inhibition of SUT genes causes severe defects in plant growth [6-9]. For example, T-DNA insertions in the Arabidopsis *AtSUC2* gene resulted in an excess of starch in source leaves, a lack of sucrose in sink tissues, and stunted plant growth [8].

According to phylogenetic analysis, plant SUTs can be grouped into three types [10,11]. Type I SUTs are only found in eudicots, and are necessary for phloem loading [6,8]. Type II transporters are present in all plants, and in monocots they are considered to function in phloem loading [9,12]. Each plant species has at least one Type III SUT, which is localized in the vacuolar membrane of cells [13-15].

Despite the importance of SUTs in plants, the substrate binding sites and transport mechanism remain largely unknown [16,17]. His65 in AtSUC1 was identified as the site of substrate-protectable modification by the inhibitor DEPC [18]. Although AtSUC1(H65C) lost sucrose transport activity, H65K and H65R exhibited higher transport rates than the wild-type [18], indicating His at this position is not essential for transport function. Charged amino acids within transmembrane spans (TMS) were identified using a 3D structural model of type II rice sucrose transporter OsSUT1 and five of them were identified as essential for sucrose transport activity [19]. Among the five amino acids, conservative mutations of Asp177, Arg188, or Asp331 resulted in complete loss of transport activity. In addition, alterations of Arg335 or Glu336 led to large decreases of the sucrose transport activity [19].

Prior to identification of the first SUT cDNA [20], substrate analogs were used as inhibitors to investigate sucrose transporter-substrate interactions using leaf discs [21], protoplasts from cotyledons [22], or plasma membrane vesicles [23]. Hydroxyls of the glucose ring are thought to be directly involved in substrate binding, while the fructosyl region provides a hydrophobic surface that is also important for binding [21,22]. Replacement of the glucosyl 4-OH or 3-OH with hydrogen or fluorine showed the most dramatic decrease in substrate recognition [21-23], indicating that the two hydroxyls interact with the SUT protein via hydrogen bonding [22]. Hydrogen substitution or fluorine substitution of the 2-OH [21,24] or 6-OH [22] also inhibited substrate transport.

Plant SUT proteins belong to the major facilitator superfamily (MFS), several members of which have been well studied [25-31]. MFS transporters share similar 3D structure [25,26,29,31,32], and operate via a “rocker-switch” mode [33,34]. The most extensively investigated MFS protein is lactose permease of *E. coli* (LacY), which transports lactose and H^+^ into the cell at a ratio of 1:1 [35]. Arg144 is one of the six irreplaceable amino acids of LacY; it is located in the middle of Helix V, facing the central cavity [26]. A substitution of Arg144 for Lys results in complete loss of lactose transport activity [36]. Arg188 of OsSUT1 has been suggested to function similarly; replacement of Arg188 by Lys results in complete loss of sucrose transport activity [19]. In LacY, Arg144 forms a bifurcated hydrogen bond with 3-OH and 4-OH groups of the galactose moiety of lactose [26,36-39]. Arg144 also interacts with Glu126 when substrates are absent, and with Glu269 during the substrate transport process [26,39].

In this paper, the role of Arg188 in the function of type II sucrose transporter OsSUT1 was further explored. The effects of additional mutations on Arg188 in OsSUT1 were tested. Since Arg188 is conserved in all SUTs, we tested the effect of mutations at this position in type I and type III SUTs. The ability of *OsSUT1* and *OsSUT1(R188K)* to rescue the dwarf phenotype of Arabidopsis *atsuc2* mutants was also tested. Fluorine derivatives of α-phenyl glucoside were used to probe the roles of hydroxyl groups at the glucosyl 3 and 4 positions in substrate-protein interactions. Based on results from these experiments we propose a putative binding interaction between Arg188 of OsSUT1 and hydroxyl groups of sucrose. A role of Arg188 in the substrate transport process is also suggested.

## Results

### Arg188 in OsSUT1 is required for transport activity

**Figure 1:**
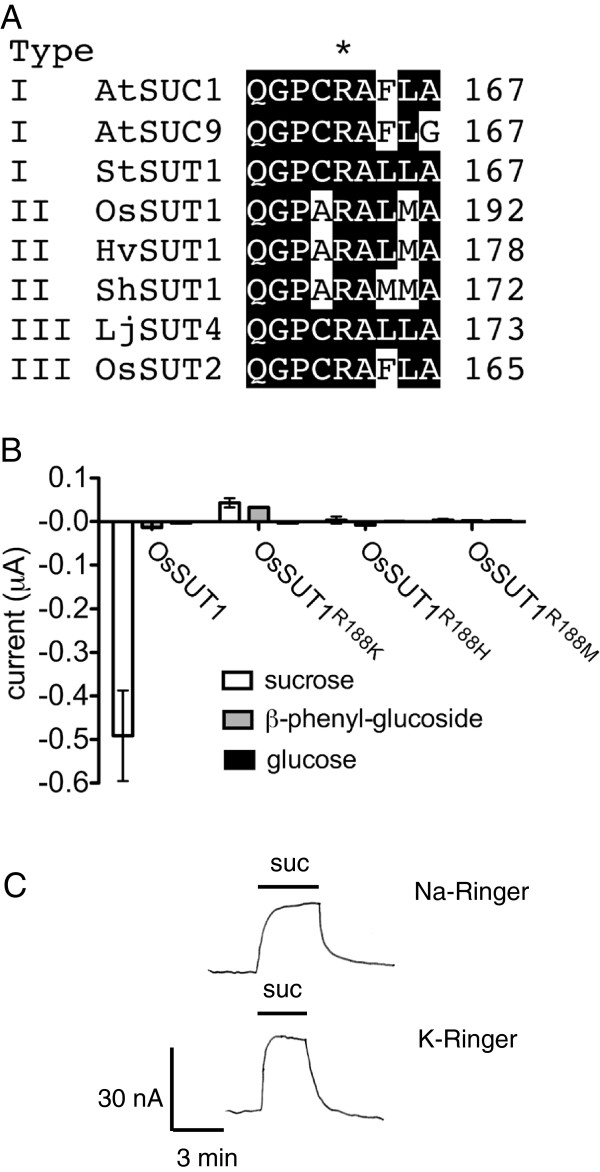
**A conserved Arg in OsSUT1 is crucial for sucrose-induced currents.** (**A**) Arg188 in OsSUT1 is conserved in all three types of SUTs. (**B**) Substrate-induced currents in oocytes expressing OsSUT1 wild type or Arg188 mutants at pH 5.6. The currents were induced by 30 mM sucrose, β-phenyl-glucoside, or glucose, at −118 mV. Error bars indicate SE (n=3). (**C**) Oocyte expressing OsSUT1(R188K) shows block of inward leak currents by 30 mM sucrose. Currents were measured in Na ringer or K ringer (Na-free) at pH 5.6, and a holding potential of −40 mV. Results in C are from one oocyte that is representative of four experiments. Pipette potential was zeroed when bath solutions were changed.

Arg188 of OsSUT1 is 100% conserved in plant SUTs (Figure 1A). The OsSUT1(R188K) mutant does not transport sucrose, yet the addition of sucrose induces a positive shift in current under voltage clamp conditions in oocytes expressing the mutant [19] (Figure 1B). β-phenyl glucoside is a substrate for Type I and III SUTs, but is not transported by Type II SUTs such as OsSUT1 [11,40]. The application of β-phenyl glucoside to oocytes expressing wild type OsSUT1 did not result in a change in current (Figure 1B). However, application of β-phenyl glucoside to oocytes expressing OsSUT1(R188K) resulted in an upward shift in currents. This shift has been previously interpreted as a substrate-dependent block of inward coupling ion (H^+^) leak [19]. In contrast, the monosaccharide glucose is not transported by SUTs, and showed no interaction with OsSUT1(R188K) mutant (Figure 1B). 

To further evaluate the role of Arg188 in OsSUT1, substitutions were made with His and Met (Figure 1B). Replacement of Arg with His retains the positive charge, while Met was selected since it has a long side chain similar in size to the side chain of Arg. Oocytes expressing R188H or R188M did not show detectable currents when sucrose was applied (Figure 1B). The upward deflection in currents was only observed for OsSUT1(R188K) and not for OsSUT1(R188H) or OsSUT1(R188M) mutants (Figure 1B). OsSUT1(R188K) localizes to the plasma membrane when expressed in oocytes [19]. The localization of R188H and R188M were not determined in oocytes, therefore the lack of transport activity could be due to transporter inactivity but we cannot rule out protein instability, degradation, or lack of targeting to the plasma membrane.

**Figure 2:**
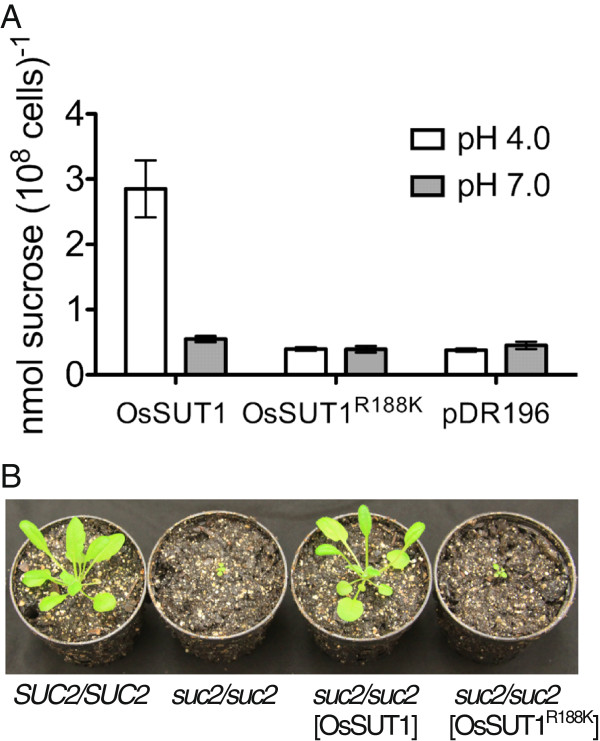
**Analysis of the function of OsSUT1 (R188K) in yeast and plants.** (**A**) ^14^C-sucrose uptake by yeast SEY6210 expressing OsSUT1 wild type, OsSUT1(R188K) mutant, and the empty vector pDR196. Assays were performed at 30°C with 1 mM sucrose in Na phosphate buffer at pH 4.0 or pH 7.0 for 5 minutes. Data are presented as mean ± SE (n=3). (**B**) 30-day Arabidopsis plants, including the wild type control (*SUC2/SUC2*), *suc2-5* homozygous (*suc2/suc2*), *suc2* homozygous transformed with *OsSUT1*, and *suc2* homozygous transformed with *OsSUT1(R188K).*

The substrate-induced upward deflection in currents were previously only measured [19] in Na ringer that contains a high concentration of Na^+^ (115mM). Therefore, experiments were performed to test whether the substrate-blocked leak current is carried by Na^+^ rather than H^+^. Oocytes expressing OsSUT1(R188K) were bathed in either Na ringer or K ringer (Na^+^-free) at pH 5.6. Application of 30 mM induced an upward current deflection consistent with block of an inward leak through OsSUT1(R188K) that was indistinguishable in either Na ringer or K ringer (Figure 1C). The results are consistent with a H^+^ leak through OsSUT1(R188K). Another possibility is that upward deflections in currents were due to sucrose/H^+^ antiport activity. OsSUT1(R188K) was previously shown to not transport sucrose at pH 4.0 when expressed in yeast [19]. We reasoned that if the R188K mutation caused a switch in OsSUT1 to antiporter activity, higher pH should stimulate ^14^C-sucrose uptake. Wild-type OsSUT1 accumulated ^14^C-sucrose to a greater extent at pH 4.0 versus pH 7.0 (Figure 2A). This is consistent with the acidic pH optimum for OsSUT1 [40] and its function as a H^+^-coupled symporter. However, OsSUT1(R188K) mutant did not show ^14^C-sucrose uptake above vector control at pH 4.0 or pH 7.0 (Figure 2A). This result favors the previous interpretation [19] that substrate block of H^+^ leak is the likely explanation for the positive shift in currents on application of sucrose or β-phenyl glucoside to oocytes expressing OsSUT1(R188K) (Figure 1B). 

### OsSUT1(R188K) does not function in plants

Loss of function mutations in the Arabidopsis sucrose transporter *AtSUC2* result in dwarf plants due to defects in carbohydrate transport in the vascular tissue [8,41]. Heterozygous *SUC2/suc2* plants do not have a visible phenotype and were transformed with either wild type *OsSUT1* or *OsSUT1(R188K)*. The constructs also contained the *AtSUC2* native promoter and its 3^’^ UTR. Transformants with the *suc2/suc2* background were identified by PCR. *OsSUT1* reversed the growth defect of the *suc2/suc2* plants, showing growth similar to wild-type *SUC2/SUC2* plants (Figure 2B). On the contrary, the *OsSUT1(R188K)* failed to rescue the *suc2/suc2* mutant (Figure 2B). The results indicate that Arg188 is necessary for transport activity in plants. Membrane localization of OsSUT1(R188K) in plants was not tested and therefore we cannot rule out the possibility that this mutant does not correctly localize to the plasma membrane.

### Arg corresponding to OsSUT1 R188 is important in type I and III SUTs

**Figure 3:**
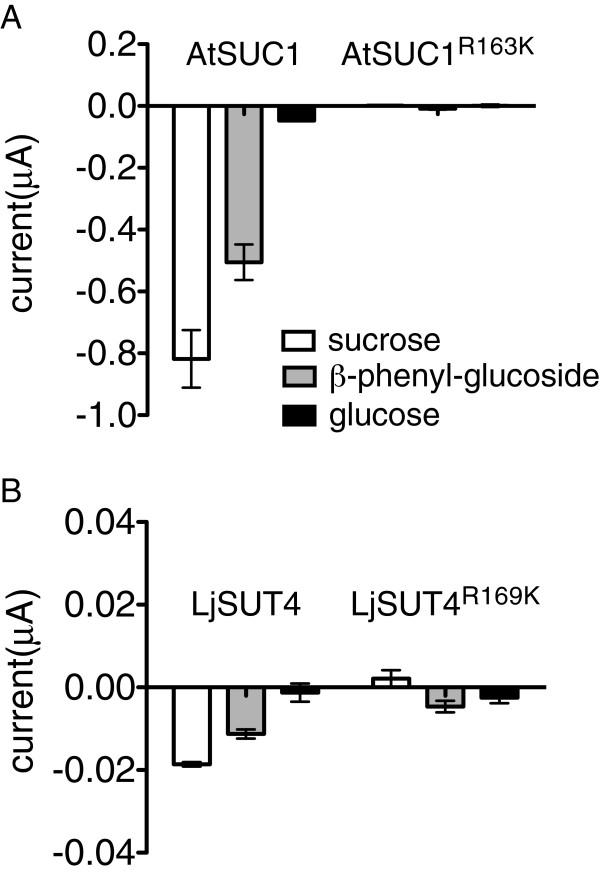
**Analysis of corresponding mutations in type I and type III SUTs.** (**A**) Substrate-induced currents in oocytes expressing type I sucrose transporter AtSUC1 wild type and AtSUC1(R163K). (**B**) LjSUT4 wild type, and LjSUT4(R169K). In A and B, currents were measured at −118 mV, pH 5.6, with a substrate concentration of 30 mM. Error bars indicate SE (n=3).

If the conserved Arg188 is important for the transport mechanism of SUTs, the corresponding Arg to Lys mutations should produce a similar defect in type I and III SUTs. The equivalent Arg in AtSUC1, a type I SUT, and LjSUT4, a representative type III SUT were mutated (Figure 1A and Figure 3). Oocytes expressing wild-type AtSUC1 displayed large inward currents when sucrose or β-phenyl glucoside were applied (Figure 3A). The AtSUC1(R163K) mutant completely lost transport activity as no inward current was observed on application of substrate (Figure 3A). Although wild-type AtSUC1 produced larger currents (−0.818 μA) than OsSUT1 (−0.491 μA) when sucrose was applied, application of sucrose to oocytes expressing AtSUC1(R163K) did not result in baseline shift of currents (Figure 3A). The results demonstrated that this conserved Arg was important for the substrate transport function of Type I SUTs.

The type III SUT LjSUT4 could transport sucrose and β-phenyl glucoside, but not glucose [15] (Figure 3B). Compared with wild-type LjSUT4, oocytes expressing LjSUT4(R169K) showed no sucrose-inducible inward currents, and no significant block of inward current by sucrose (Figure 3B). As the sucrose-induced inward current of LjSUT4 (−0.019 μA) was 26 times smaller than that of OsSUT1 (−0.491 μA), a block of an inward H^+^ leak in LjSUT4(R169K) may have been too small to measure. However, the results supported the conclusion that the conserved Arg was crucial for the substrate transport in Type I, II, and III SUTs.

### Hydroxyls 3 and 4 in the glucose moiety of sucrose are crucial for substrate transport

**Figure 4:**
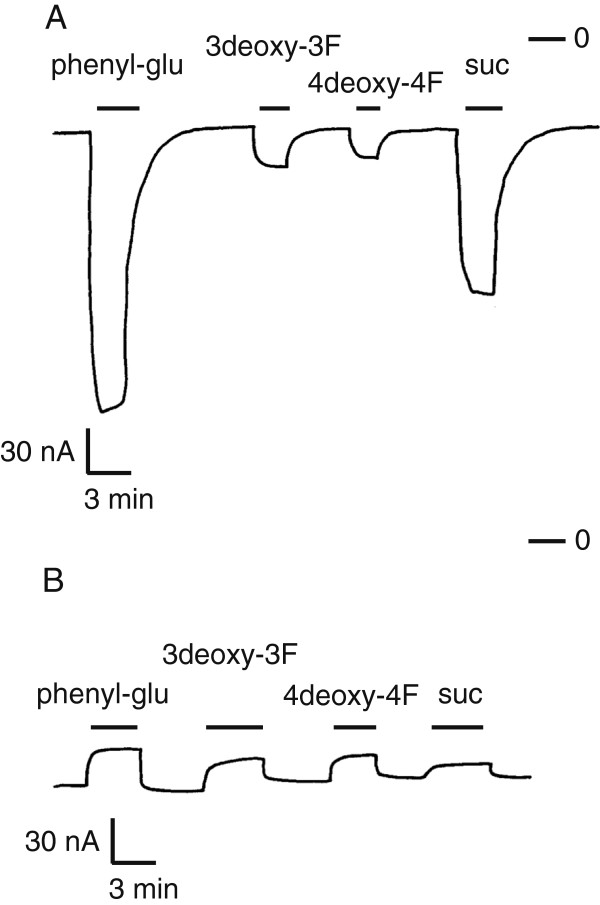
**Currents induced by deoxy-fluoro derivatives of α-phenyl glucoside in oocytes expressing OsSUT1 wild type or OsSUT1(R188K).** (**A**) Xenopus oocytes expressing OsSUT1 wild type were voltage clamped at -40mV in Na ringer solution at pH 5.6. Currents were recorded in response to application of the following substrates at 30 mM: α-phenyl glucoside (phenyl-glu), phenyl-3-deoxy-3-fluoro-α-glucoside (3deoxy-3F), phenyl-4-deoxy-4-fluoro-α-glucoside (4deoxy-4F), and sucrose (suc). Zero current is indicated (−0). (**B**) Currents recorded in oocytes expressing OsSUT1(R188K) in response to the same substrates and using the same conditions as in A.

In early investigations regarding the binding sites of SUTs, deoxyl analogs or deoxy-fluoro derivatives of substrates were used to inhibit the transport of ^14^C-sucrose [21-23]. It was not clear, however, whether the analogs could be transported by SUTs. Two deoxy-fluoro derivatives of α-phenyl glucoside were used in oocyte electrophysiology experiments, because the size of fluorine is very similar to the size of a hydroxyl group and organic fluorine does not participate in hydrogen bonds as a donor or acceptor [42]. α-phenyl glucoside is transported by SUTs and it induces larger currents than sucrose in oocytes [40] (Figure 4A). A single substitution of either the 3-OH or 4-OH of α-phenyl-glucoside for a fluorine (−F) caused large decreases of currents in OsSUT1 (Figure 4A). This decrease of transport caused by deoxy-fluoro substitution supported the previous findings that 3-OH and 4-OH of substrates are important for substrate binding by SUTs. 

For oocytes expressing OsSUT1(R188K), an upward deflection of current was observed in the presence of α-phenyl glucoside (Figure 4B). This indicated that α-phenyl glucoside interacted with the mutant transporter and blocked the H^+^ leak (Figure 4B). This interaction is likely to be an initial recognition step between potential substrates and SUT proteins, because it occurred between β-phenyl glucoside and OsSUT1(R188K) (Figure 1B). When phenyl-3-deoxy-3-fluoro-α-glucoside or phenyl-4-deoxy-4-fluoro-α-glucoside was applied to oocytes expressing OsSUT1(R188K), a decrease of inward current was again observed (Figure 4B). The two deoxy-fluoro analogs were transported at a lower rate than sucrose by wild-type OsSUT1 (Figure 4A). However, they had equal or better H^+^ blocking effect compared to sucrose in OsSUT1(R188K) mutant (Figure 4B). This indicated that the 3-OH and 4-OH were more important for the substrate transport step than for the initial substrate recognition step.

## Discussion

The conserved Arg188 of OsSUT1 was previously suggested to be an essential amino acid for substrate transport by SUTs [19]. Here we show that substitution of Arg188 for Lys, His, or Met resulted in loss of substrate-inducible inward currents when expressed in oocytes (Figure 1B). Mutations of the corresponding Arg in Type I and Type III SUTs also showed a complete loss of substrate-inducible inward currents (Figure 3). When expressed in Arabidopsis under *AtSUC2* native promoter, *OsSUT1* reversed the dwarf phenotype of Arabidopsis *atsuc2* mutant but *OsSUT1(R188K)* did not. These results further support the suggestion that Arg188 of OsSUT1 is essential for transport activity.

### Putative binding interactions between Arg188 and sucrose

The sucrose-induced upward deflection in currents observed in oocytes expressing OsSUT1(R188K) (Figure 1B, 1C and 4B) is interesting because it shows that the mutant retains the ability to bind sucrose. However, as shown by expression in yeast and ^14^C-sucrose uptake experiments, OsSUT1(R188K) does not transport sucrose across the membrane [19]. Lack of ^14^C-sucrose uptake activity at pH 4.0 or 7.0 supports the idea that OsSUT1(R188K) has a H^+^ leak that is blocked by sucrose rather than sucrose/H^+^ antiport activity.

There is evidence that substrate binding involved in blocking the H^+^ leak is different than substrate binding required for glucoside translocation. First, the mutant OsSUT1(R188K) retains substrate binding but does not translocate substrates. Second, β-phenyl glucoside blocks the H^+^ leak but is not translocated by wild-type OsSUT1. Third, β-paranitrophenyl glucoside inhibits sucrose transport activity of type II SUT from barley, HvSUT1, but is not a translocated substrate [43]. There is also evidence that a H^+^ leak in the absence of substrates occurs in wild-type sucrose transporters [4] but at a lower rate. For example, sucralose acts as a competitive inhibitor of a type II SUT from sugarcane, ShSUT1, but sucralose application alone does not cause an upward shift in currents in ShSUT1-expressing oocytes [44]. We hypothesize that substrate binding that blocks H^+^ leak is preliminary to substrate binding required for translocation.

Electrophysiological assays using deoxyl-fluoro derivatives showed that the 3-OH and 4-OH of substrates were more important for the substrate transport process than the initial substrate recognition step (Figure 4). Similarly, the interaction between potential substrates and OsSUT1(R188K) suggested that Arg188 of OsSUT1 was more important for substrate transport than for initial substrate recognition. Therefore, it is reasonable to hypothesize that Arg188 of OsSUT1 interacts with 3-OH and 4-OH of the substrate during the transport process.

Arg188 in OsSUT1 appears to have a similar function as Arg144 in LacY. Arg144 of LacY is the only positively charged key amino acid in the N-terminal half of the transporter [26,27] and it is located in the middle of Helix V, one of the helixes facing the central cavity [26,39]. The LacY(R144K) mutant has no lactose transport activity [36], demonstrating that both guanidine groups -NH_2_ are crucial. Similarly, Arg188 of OsSUT1 is the only positively charged key amino acid identified in the N-terminal half of the transporter [19]. In OsSUT1, Arg188 is predicted to be located in the middle of Helix IV that surrounds the central transport pathway [19]. The OsSUT1(R188K) mutant does not transport sucrose [19] (Figure 1B, 2, 4B), indicating that both -NH_2_ of this Arginine are essential.

**Figure 5:**
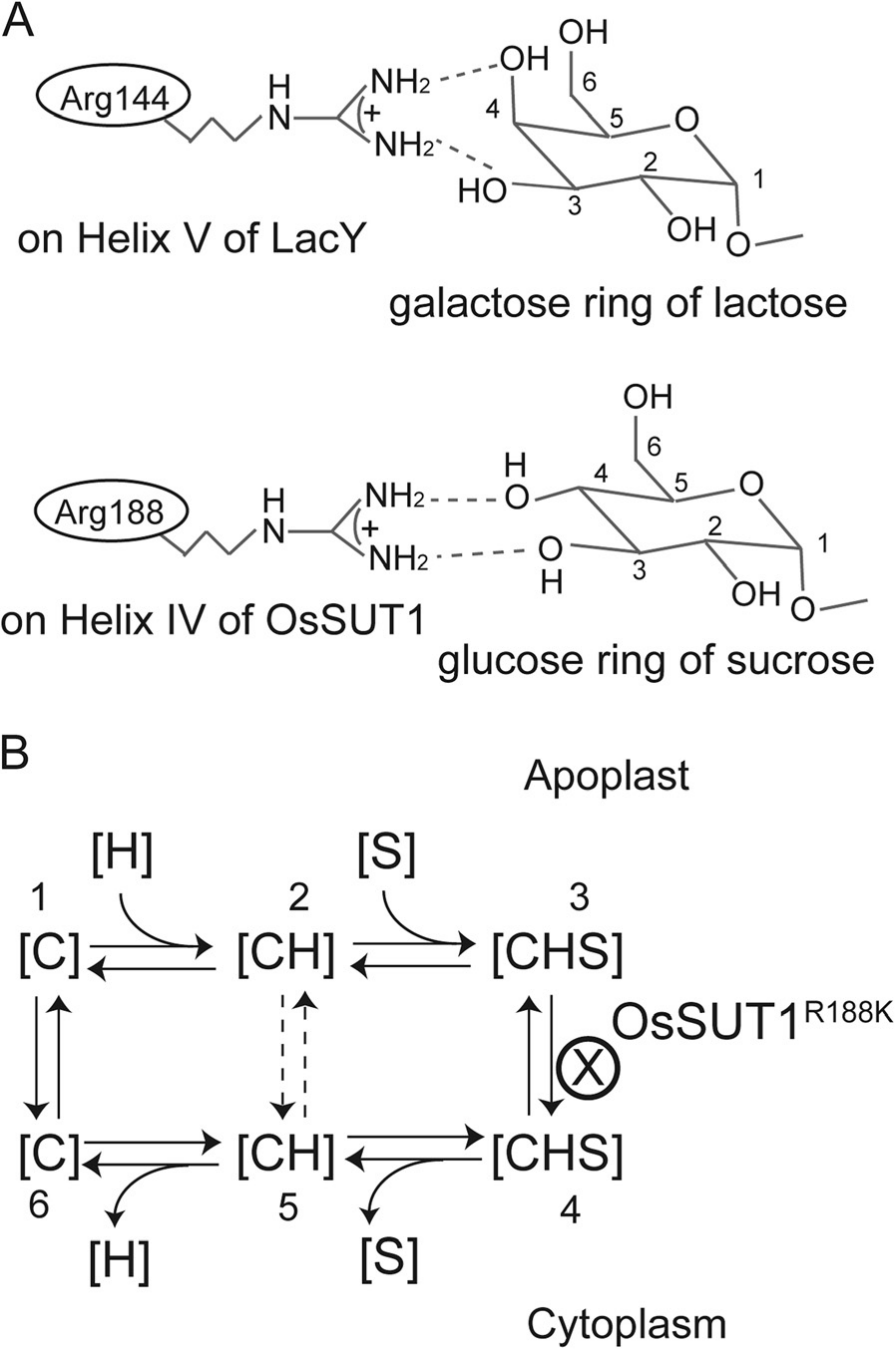
**Model for the function of Arg188 in OsSUT1.** (**A**) Upper: known interaction between Arg144 of LacY and the galactose ring of lactose [38]. Lower: Suggested interaction between Arg188 of OsSUT1 and the glucose ring of sucrose. (**B**) Kinetic model for a SUT protein. C represents the SUT protein, S represents sucrose, and H is the proton. The dashed arrows between the two [CH] statuses indicate H^+^ flows across through SUTs without transporting sucrose. In the OsSUT1(R188K) mutant, sucrose binds to the transporter from the apoplastic side, but cannot be released to the cytoplastic side, keeping the mutated protein in the [CHS] apoplastic status (step 3).

The interaction of OsSUT1 Arg188 with its substrate is modeled after the well-studied interaction of Arg 144 in LacY with lactose (Figure 5A). A bidentate hydrogen bond between NH_2_ groups of Arg188 in OsSUT1 and 3-OH and 4-OH groups of sucrose is proposed. For LacY, the 2, 3, 4, and 6 hydroxyl groups –OH in the galactose ring of lactose, especially 3-OH and 4-OH, are important for the substrate binding and transport [37,38]. Similarly, the 2, 3, 4, and 6 hydroxyl groups in the glucose moiety of sucrose, particularly 3-OH and 4-OH, are essential for the substrate recognition and transport in SUTs [21-24]. Further work will be required to determine whether one of the NH_2_ groups of Arg188 interacts with another amino acid in the N-terminal half of protein, which triggers a major conformational change (“rocker-switch”) analogous to the mechanism in LacY [33]. 

### OsSUT1(R188K) in the sucrose transport process

All previously proposed transport mechanisms for SUTs are in agreement that on the cytoplasmic side of the membrane, sucrose leaves the SUT protein before release of the proton [4,17,45]. However, one model involves a sequential loading of the transporter on the extracellular side with proton binding followed by sucrose [4,17]. Other results support a random binding model in which either sucrose or a proton can bind first on the extracellular side [45]. Our results support an ordered binding of protons followed by sucrose (Figure 5B). In this model, the transporter binds a proton on the extracellular side of the membrane and this facilitates sucrose binding (Figure 5A, stages 1–3). The fully loaded SUT transporter has a conformational change, termed the “rocker-switch” [33], to face the cytoplasmic side (Figure 5B, stage 3–4). Sucrose and proton are then released (Figure 5B, stage 4–6). The empty carrier then flips back, returning its substrate binding sites to the apoplastic side (Figure 5B, stage 6–1). Some protons bound at the apoplast of a SUT could be released directly to cytoplasm (Figure 5, stage 2–5), bypassing the sucrose-binding process. The un-coupled transport of H^+^ has been observed in wild-type StSUT1, but the rate was lower than the H^+^-coupled transport of sucrose [4].

The OsSUT1(R188K) mutant has a larger uncoupled H^+^ leak, compared to wild-type OsSUT1, that is blocked by substrate (Figure 4B). However R188K does not transport sucrose. Therefore, it is likely that R188K is blocked just prior to the “rocker switch” step in the transport cycle (Figure 5B, stage 3). Substrate binding inhibits uncoupled transport (Figure 5B, stage 2–5), most likely by progressing the transporter to stage 3 (Figure 5B) where the cycle is blocked. This explanation supports the concept that Arg188 of OsSUT1 is essential for the transport of substrates across the membrane at the “rocker-switch” step (Figure 5B, stage 3–4).

## Conclusions

R188 in OsSUT1 was identified as a charged amino acid within the fourth TMS that is important for transport activity [19]. The R188K mutation in OsSUT1 results in a lack of sucrose transport but when assayed by voltage clamping in oocytes, an upward deflection in current occurs when sucrose is applied. The previous suggestion that this represents a substrate-induced block of H^+^ current [19] appears to be correct. The inward leak though R188K is independent of Na^+^ (Figure 1C) and R188K does not appear to function as an antiporter (Figure 2A). We also show that while OsSUT1 is functional in the phloem when expressed in Arabidopsis *atsuc2-5*, the R188K mutant is not. This amino acid position is also conserved in type I and type III SUTs (Figure 1A) and corresponding mutations in Arabidopsis AtSUC1 and *Lotus japonicus* LjSUT4 resulted in a loss of transport activity (Figure 3). Deoxy-fluoro derivatives of α-phenyl glucoside were used to investigate substrate binding by OsSUT1. Modification at the glucosyl 3-OH and 4-OH positions significantly reduced transport by wild type OsSUT1. However, substrate-induced leak current block observed in the OsSUT1(R188K) mutant was not affected. Based on these results, we propose 1) that in OsSUT1, R188 is involved in substrate translocation and 2) an additional substrate binding site, independent of R188, functions in initial substrate recognition and block of H^+^ leak through the R188K mutant.

## Methods

### Constructs for oocyte experiments

Mutagenesis of *OsSUT1* in pCR8/GW was performed using the QuikChange II site-directed mutagenesis kit (Stratagene). PCR reactions included dimethyl sulfoxide (DMSO) at a final concentration of 8% to inhibit the formation of secondary structure. The *pCR8/GW-OsSUT1(R188H)* or *pCR8/GW-OsSUT1(R188M)* were recombined with oocyte vector pOO2/GW. *AtSUC1(R163K)* and *LjSUT4(R169K)* mutants were made using *AtSUC1* and *LjSUT4* constructs in the POO2/GW vector using the QuikChange II site-directed mutagenesis kit without the addition of DMSO. All sequences were confirmed. cRNAs were prepared using the SP6 mMessage mMachine kit (Ambion). Oocyte preparation and two-electrode voltage clamp recordings (TEVC) were the same as previously described [43]. Oocytes were bathed in modified Na ringer solution (115 mM NaCl, 2.5 mM KCl, 1.8 mM CaCl_2_, 1 mM NaHCO_3_, 10 mM MgCl_2_, 10 mM MES-Tris, pH 5.6) or K ringer (115 mM KCl, 1.8 mM CaCl_2_, 1 mM NaHCO_3_, 10 mM MgCl_2_, 10 mM MES-Tris, pH 5.6).

### ^14^C-sucrose uptake

Yeast (*Saccharomyces cerevisiae*) strain SEY6210 (Matα ura3-52 leu2-3, 112 his3-Δ200trp1-Δ901 lys2-801 suc2-Δ9) [46] transformed with *pDR196/GW-OsSUT1*, *pDR196/GW-OsSUT1(R188K)*, or empty vector *pDR196/GW* was used for ^14^C-sucrose uptake experiments as previously described [19]. Uptake assays were done at pH 4.0 or 7.0, and SEY6210 cells were incubated in 1 mM sucrose for 5 minutes at 30°C. They were then washed three times using ice-cold 10 mM sucrose, and radioactivity was counted.

### Arabidopsis atsuc2 complementation

Constructs for plant transformation were made by assembling the *AtSUC2* (At1g22710) promoter in pDONR P4-P1R [11], the ORF of *OsSUT1* or *OsSUT1(R188K)* in pCR8/GW [19], and the 3’ UTR of *AtSUC2* in pDONR P2R-P3 [11] into the pB7m34/GW binary vector [47]. LR Clonase Plus (Invitrogen) was used for this directional three-fragment recombination. *Agrobacterium tumefaciens* strain C58C1 containing the constructs was used to transform Arabidopsis *atsuc2-5* heterozygous plants (SALK_087046) [48]. Basta-resistant transformants were selected on soil, and homozygous *atsuc2-5* lines were identified via PCR.

### Deoxy-fluoro derivatives

Phenyl-3-deoxy-3-fluoro-α-D-glucoside and phenyl-4-deoxy-4-fluoro-α-D-glucoside were supplied by Carbosynth Limited (Berkshire, UK).

## Authors’ contributions

YS and JMW designed the study. YS performed the experiments. Results were evaluated and the manuscript was written by YS and JMW. All authors read and approved the final manuscript.
